# Mouse newborn cells allow highly productive mouse cytomegalovirus replication, constituting a novel convenient primary cell culture system

**DOI:** 10.1371/journal.pone.0174695

**Published:** 2017-03-24

**Authors:** Vu Thuy Khanh Le-Trilling, Mirko Trilling

**Affiliations:** Institute for Virology, University Hospital Essen, University Duisburg-Essen, Essen, Germany; University of St Andrews, UNITED KINGDOM

## Abstract

Mammalian cell culture is indispensable for most aspects of current biomedical research. Immortalized cell lines are very convenient, but transforming principles (e.g. oncogenic viruses or their oncogenes) can heavily influence the experimental outcome. Primary cells do not share this apparent disadvantage but are more laborious to generate. Certain viruses (e.g. mouse cytomegalovirus) do not replicate efficiently in most transformed cell lines. In the past, such viruses have been routinely propagated on primary mouse embryonic fibroblasts (MEF) established around day 17 (d17) of gestation. According to new regulations of the European Union, experiments using gravid mammals and/or their embryos in the last trimester (>d14 in the case of mice) of gestation do require explicit permission of the local authorities responsible for animal care and use. Applying for such permission is time-consuming and often inflexible. Embryonic fibroblasts could also be produced at earlier time points of pregnancy from younger and smaller embryos. Obviously, this approach consumes more pregnant mice and embryos. Newborn mice are larger thus yielding more cells per sacrificed animal and the new Directive (2010/63/EU) excludes the killing of animals solely for the use of their organs or tissues. We established a convenient protocol to generate adherent mouse newborn cells (MNC). A direct comparison of MNC with MEF revealed that MNC fully recapitulate all tested aspects of a broad panel of virological parameters (plaque size, final titers, viral replication kinetics, viral gene expression, drug and interferon susceptibility as well as species specificity). The herein described approach allows researchers the legal use of primary cells and contributes to the 3R (replace, reduce, refine) guiding principles—especially the ‘reduce’ aspect—for the use of animals in scientific research. Additionally, it offers the option to directly compare *in vitro* and *in vivo* experiments when MNC are generated from littermates of animals included in the *in vivo* experiments.

## Introduction

Most aspects of current biomedical research require mammalian cell culture experiments. Even researchers who mainly focus on *in vivo* experiments often need mammalian cell cultures e.g. for mechanistic studies, to propagate pathogens for infection experiments or to generate certain biomolecules like growth factors. Cell cultures using previously established cell lines with immortal replication capability do not require use of animals—maybe with the notable exceptions of animal sera routinely added to cell culture media. However, the transforming principles (e.g. oncogenic viruses or their respective oncogenes) can significantly influence the outcome of experiments. In this respect, viral transformation can influence e.g. the cell cycle regulation, apoptosis induction as well as interferon induction and signalling. The famous HEK 293 cells harbour an insertion of 4344 nucleotides derived from Adenovirus 5 comprising the coding sequence for the viral proteins E1a and E1b [1] which interact with numerous cellular proteins and affect several important cellular events. The HEK 293-derived 293T cells additionally express the SV40 Large T antigen. These aberrations of cell lines are exemplified by the fact that Leigh van Valen, famous for his formulation of the red queen hypothesis and the law of extinction, proposed that HeLa cells even deserve a rank of a separated species—termed *Helacyton gartleri* [2]—based on profound differences between HeLa cells and primary human tissue.

Certain viruses (e.g. mouse cytomegalovirus [MCMV; Murid herpesvirus 1, TaxID 10366]) are very restrictive and only replicate efficiently in a few immortal cell lines like NIH/3T3 cells (ATCC^®^ CRL-1658^™^). High titer stocks are therefore routinely produced on primary mouse embryonic fibroblasts (MEF) which are established from *Mus musculus* embryos at or around day 17 to 18 (d17-18) of gestation [3]. ‘*Although alternatives are available (e*.*g*., *immortalized fibroblast cell lines)*, *MEFs still represent the gold standard for virus propagation and titration*’ [3].

MCMV is a very well established model system to study the pathogenesis of cytomegaloviruses *in vitro* and *in vivo*. Due to the strict species-specificity of cytomegaloviruses, it is not possible to conduct meaningful *in vivo* experiments with human CMV in small animal models (e.g. in mice or rats). To overcome this hurdle, related cytomegaloviruses (e.g. mouse, rat, guinea pig and rhesus CMV) are used in their respective host species as model systems. MCMV infections of mice constitute one of the few infection models in which a DNA virus can be studied in its native host species. Consistently, MCMV is also frequently used in basic immunological research.

Recently, the European Union (EU) has revised the regulations concerning animal experiments (see Directive 2010/63/EU). This amendment covers foetal forms of mammals if the developmental forms are allowed to live beyond the first two thirds of their development. Accordingly, experiments using gravid mammals and/or their embryos in the last trimester of gestation require by definition the explicit permission from the responsible committees for Animal Care and Use. This EU law has meanwhile been implemented in the respective legislations of the EU member states. Although the US Animal welfare act does not cover rodents and birds, the Public Health Service Policy requests the usage of the minimum number of animals required to obtain valid results and the minimization of discomfort, distress, and pain when consistent with sound scientific practices.

The application process for a permission to prepare embryonic fibroblasts is time-consuming and does not confer much flexibility e.g. new mouse lines (like genetically modified ones) cannot be easily integrated and have to be registered individually. An apparent design-around strategy would be to use pregnant mammals and/or their embryos before the last trimester—in the case of mice before day 14. But at this time of ontogeny, embryos are smaller. Therefore, more animals have to be sacrificed to prepare a given number of cells. This approach would obviously corrupt the intention of above mentioned amendment of the regulations concerning animal experiments. Additionally, sometimes gravidity cannot be judged with absolute certainty. Thus, a number of animals are unnecessarily sacrificed for the sake of generating embryonic tissues, organs or cells because the animals are actually not pregnant. This applies especially to mouse lines which breed poorly, are obese or have only few or smaller embryos.

All limitations mentioned above do not apply to mice which have been born. The directive 2010/63/EU and its regional counterparts explicitly excluded ‘*the killing of animals solely for the use of their organs or tissues*’. Consistently, vertebrates can be sacrificed to generate cells or tissues for research purposes without specific permission. More importantly, newborn mice are larger than embryos and yield far more cells. In the case that primary cells are required, the use of newborn cells instead of embryonic fibroblasts would reduce the number of animals needed for a given experiment. Obviously, the issue of pseudo-pregnancy or uncertainty concerning pregnancy is also circumvented.

Therefore, we have established an easy and convenient protocol to generate adherent fibroblasts from newborn mice. A direct side-by-side comparison between primary MEF and primary MNC revealed virtually congruent characteristics in all tested aspects of MCMV research suggesting that MNC and MEF can be used interchangeably.

## Results

### The overall morphology as well as the MCMV-induced plaque formation of MEF and MNC are almost indistinguishable

**Fig 1:**
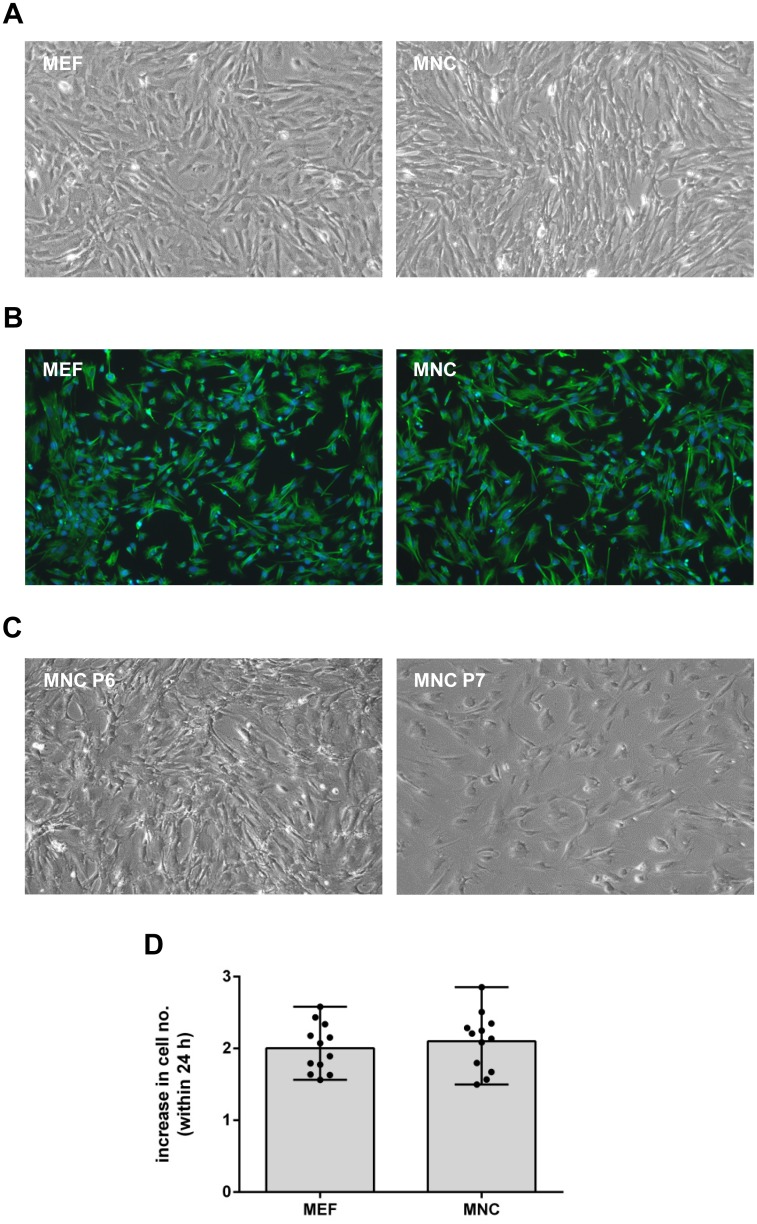
(A) Primary MEF and MNC were prepared and expanded for experimental use (passage 3). Cell morphology was regularly examined and compared. Representative bright-field microscopy images of both cell types are shown. (B) Passage 3 MEF and MNC were fixed with 4% (w/v) paraformaldehyde and permeabilized with 0.05% (v/v) Triton-X. For visualisation of vimentin, an AlexaFluor488-coupled antibody (Abcam) was used for immunofluorescence staining (shown in green). Nuclei were stained with DAPI (shown in blue). (C) MNC were propagated beyond passage 3. Bright-field microscopy images of passage 6 and passage 7 cells are shown. (D) To analyse the cell growth, the increase in cell number within 24 h of proliferation during the exponential phase was determined. Dots depict values of individual measurements. Bars depict the mean with range.

We established a protocol for the preparation of MNC. MEF and MNC grew as adherent cultures with contact inhibition leading to homogenous cell monolayers with highly similar overall appearance (see Fig 1A and data not shown). To evaluate the cell type and purity of the primary MEF and MNC cultures, we visualized vimentin by immunofluorescence staining. Vimentin is a marker for mesenchymal cells and is expressed in fibroblastic cells. Vimentin is also present in many other cell types than fibroblasts, though in a lower level. Since, to our knowledge, no definitive marker for fibroblasts exits, we judged a strong vimentin staining together with the spindle-shaped morphology as hallmark for fibroblasts. Fig 1B shows that virtually all MEF as well as MNC meet this criterion. The quantification by flow cytometry revealed that 97% of the MEF and 99% of the MNC cultures were positive for vimentin (data not shown). To test how often primary MNC can be passaged beyond passage 3, we propagated the cells until signs of senescence appeared. In passage 5, the growth was decelerated (data not shown). In passage 6, an altered morphology was observable. Senescence became evident in passage 7 (Fig 1C). In addition, we compared the cell replication rates of MEF and MNC. The determination of cell numbers during 24 h of exponential proliferation revealed that MEF cell numbers increased 2.00 +/- 0.43 fold and MNC cell numbers 2.1+/-0.75 fold (Fig 1D).

**Fig 2:**
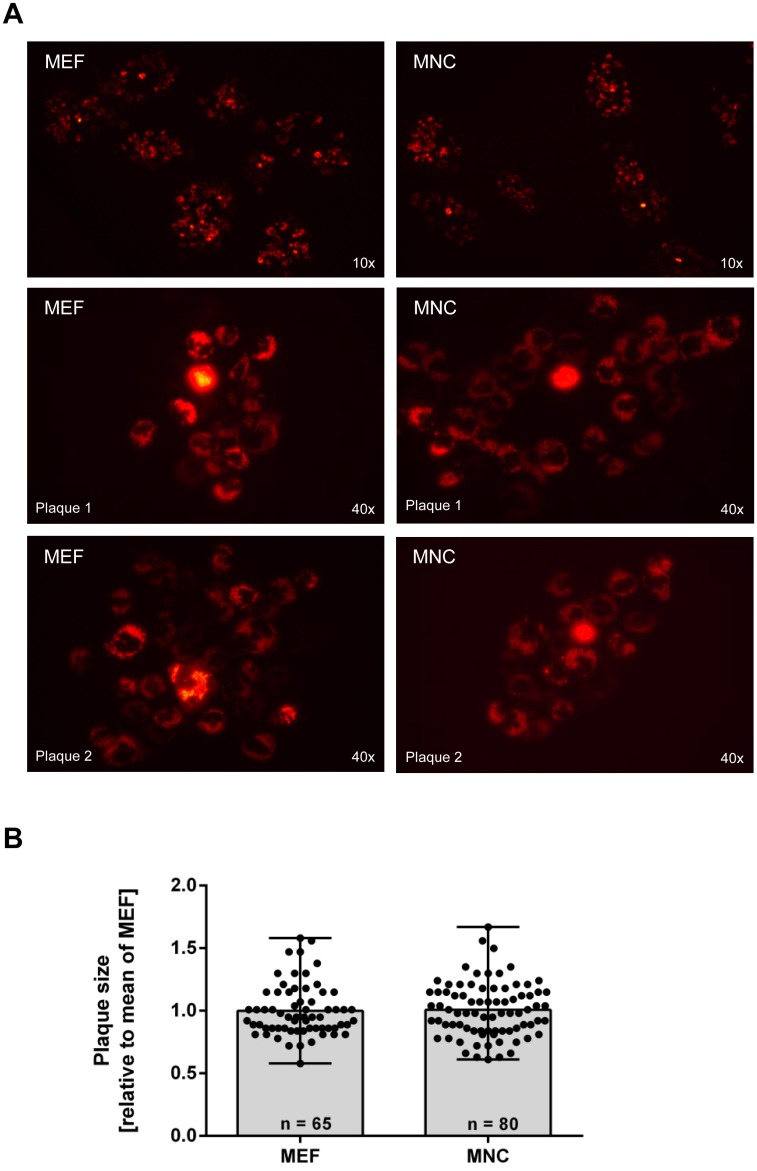
(A) MEF and MNC were infected with Δm157-MCMV:dsRedMito (MOI 0.01). Supernatant spread to neighboring cells was limited by methylcellulose overlay. At 3 days post infection, reporter gene expression was monitored by fluorescence microscopy. (B) Relative plaque size of Δm157-MCMV:dsRedMito-infected MEF and MNC. Plaque diameters were determined at 3 days post infection (methylcellulose overlay). Dots depict values of individual plaques. Bars depict the mean with range.

To compare the permissiveness of MNC and MEF for MCMV, cells were infected with low multiplicities of infection (MOI) and covered with methylcellulose containing medium to ensure plaque formation and to prevent virus dissemination within the cell culture dish via the medium. An MCMV mutant expressing the fluorescent protein dsRed fused to the mitochondrial targeting sequence of human cytochrome c oxidase subunit VIII (called *dsRed-Mito*) [4] was used to visualize infected cells. MCMV:*dsRed-Mito* turns mitochondria of infected cells red fluorescent, thereby generating a specific staining pattern. Additionally, if cells are lysed (e.g. in abortive or lytic infection events), the stained mitochondria are released and mark the area in which the cells was situated previously (data not shown). However, we did not observe abortive infection events—as would have been indicated by cell-free dsRed-stained mitochondria—in MEF or MNC cultures (data not shown). In most plaques, one very bright cell was evident in the middle of the plaque surrounded by cells which are dsRed-positive but less bright (see Fig 2A—lower panel). This is consistent with the interpretation that the bright cell constitutes the primarily infected cell and the surrounding cells have been infected in the second round of virus replication. The number of plaques per well, their appearance as well as the dimension of individual plaques were indistinguishable between MEF and MNC (Fig 2A and 2B).

### MEF and MNC exhibit highly similar kinetics and patterns of viral gene expression

**Fig 3:**
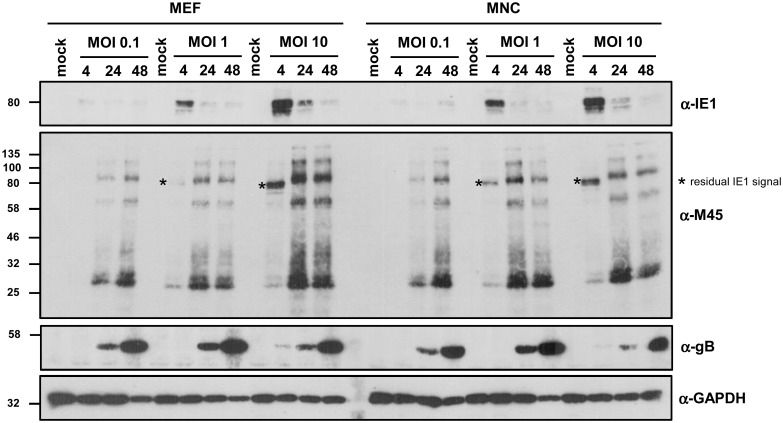
MEF and MNC were infected with Δm157-MCMV:luc (MOI as indicated) or were mock treated. At 4, 24 and 48 h post infection, protein lysates were generated. Viral gene expression was analysed by immunoblot analysis using MCMV-specific antibodies.

MEF and MNC were infected with 0.1, 1 and 10 PFU/cell, respectively, or left uninfected (‘mock’). After 4, 24 or 48h post infection, cells were lysed, lysates were normalized according to the protein concentration and subjected to immunoblot analysis using pIE1/pp89-, pM45- and pM55/gB-specific antibodies. The detection of GAPDH served as loading control. The pIE1 protein is expressed with *immediate early* kinetics [5], pM45 is expressed with *early* kinetics (but it is also incorporated into the virion) [6] and gB is expressed with true *late* kinetics [7]. The detection of these three MCMV-encoded proteins serves as surrogate test for the three major classes of cytomegaloviral gene/protein expression. While the pIE1-specific antibody stained one specific band at the expected size, the antibody against pM45 (previously described in [8]) detected more than one viral protein. Transfection of a pM45 expression plasmid into uninfected cells resulted in the same immunoblot signals as observed in infected cells (data not shown), suggesting that different isoforms or posttranslational modifications of pM45 exist. This finding might e.g. be relevant, if foreign epitopes are inserted into the *M45* sequence [9]. Across all MOIs and time points, the pattern and band intensity of viral proteins was highly similar between MEF and MNC (Fig 3). Only after infection with MOI 10 and at late time points, the intensity of M45 signals appeared to be slightly different in MEF and MNC. When the respective condition was reassessed in a repetitive manner and bands from five replicates each were quantified, this trend prevailed but did not fully meet significance criteria (p = 0.0534; data not shown). However, based on the occurrence of late gene expression and viral plaque formation, we concluded that early gene expression occurs sufficiently.

### Identical MCMV replication kinetics and Ganciclovir susceptibility in MEF and MNC

**Fig 4:**
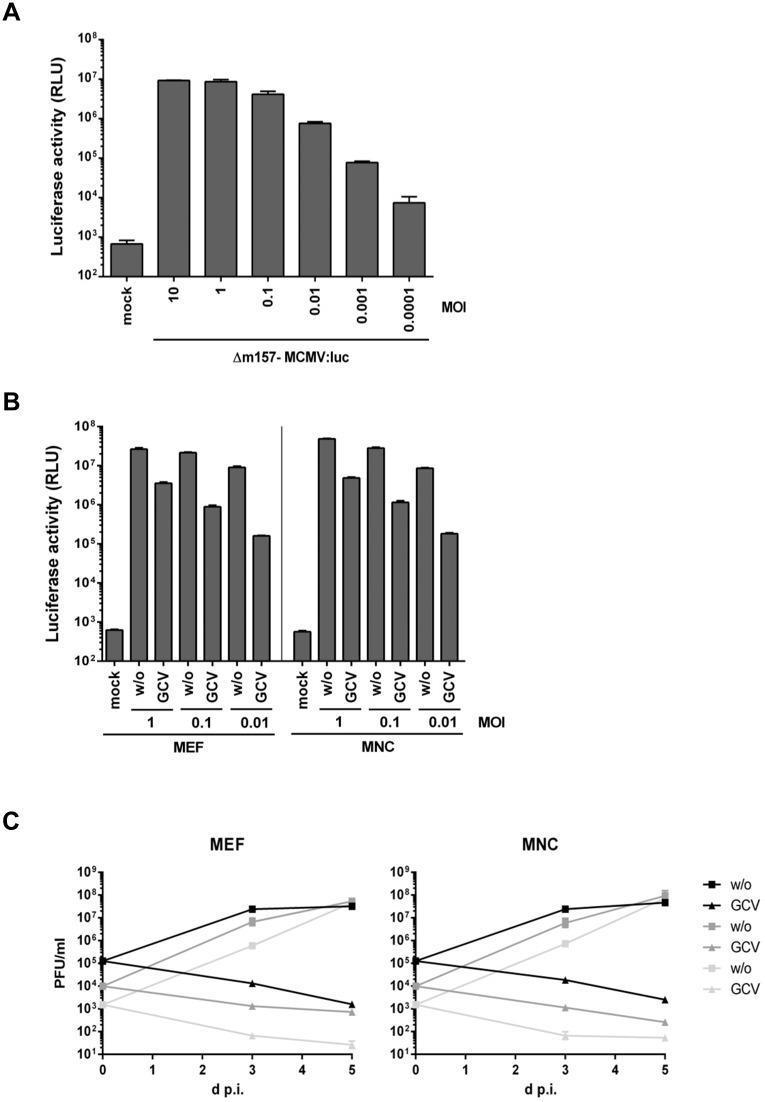
(A) To test the dose response of the luciferase reporter gene expression by Δm157-MCMV:luc, MNC were infected with grading virus doses. At 48 h post infection, cells were lysed and luciferase activity was determined. Each condition was measured in triplicate. Bars depict mean values +/- standard deviation (SD). (B) MEF and MNC were infected with Δm157-MCMV:luc (indicated MOI) in the absence and presence of ganciclovir (50 μM). At 3 and 5 days post infection, cells were lysed and luciferase activity was determined. The data for 3 days post infection are shown. The results for 5 days post infection are similar (data not shown). Each condition was measured in triplicate. Bars depict mean values +/- SD. (C) The supernatants of the ganciclovir experiment in (B) were collected and frozen before the cells were lysed. Supernatant virus was quantified by plaque titration on MEF. Each titration was done in triplicate. Mean values +/- SD are shown.

To determine the correlation between reporter gene expression and viral replication, MNC were infected with grading infectious doses (ranging from 0.0001 to 10 PFU/cell) of an MCMV (reporter-) virus expressing the luciferase gene derived from the firefly *Photinus pyralis* under the control of the *m157* promoter-enhancer [10]. *m157* is expressed with *early* expression kinetics [11]. At 48 h post infection, the cells were lysed and luciferase activity was quantified. An obvious dose-response relation which saturated at MOIs exceeding 1 was observed (Fig 4A), indicating that luciferase activity can be used as surrogate test for viral titers, and highlighting the dynamic range of the assay. MEF and MNC were infected with 0.01, 0.1 and 1 PFU/cell, respectively, or left uninfected. Cells were either treated with Ganciclovir (GCV) or the solvent. After 3 days of infection, cells were lysed and luciferase activity was quantified. GCV significantly reduced but did not abrogate virus-encoded luciferase activity (Fig 4B). GCV blocks viral genome replication and affects all subsequent steps that depend on genome amplification e.g. *late* gene expression and virus dissemination. The difference between GCV and untreated cells in terms of luciferase activity was more apparent in the low MOI infections (Fig 4B). Due to the *early* expression kinetics of *m157* and its replacement luciferase, infected cells express luciferase protein even if viral replication is prevented by GCV at the level of genome replication. At low MOI infections, the contribution of the initial virus inoculum to the final reporter gene expression is obviously lower compared to high MOI infections. Therefore, the effect of GCV is more pronounced in low MOI infections (Fig 4B). Nevertheless, in all settings, the luciferase activity was congruent between MEF and MNC.

To determine the viral replication and the GCV susceptibility, the supernatants of above mentioned experiment were collected and the infectious virus progeny was determined by classic plaque titration. GCV potently inhibited MCMV replication in MEF and MNC. MCMV replication in the absence of GCV and the GCV susceptibility was almost identical in MEF and MNC (Fig 4C) highlighting the suitability of MNC for MCMV research.

**Fig 5:**
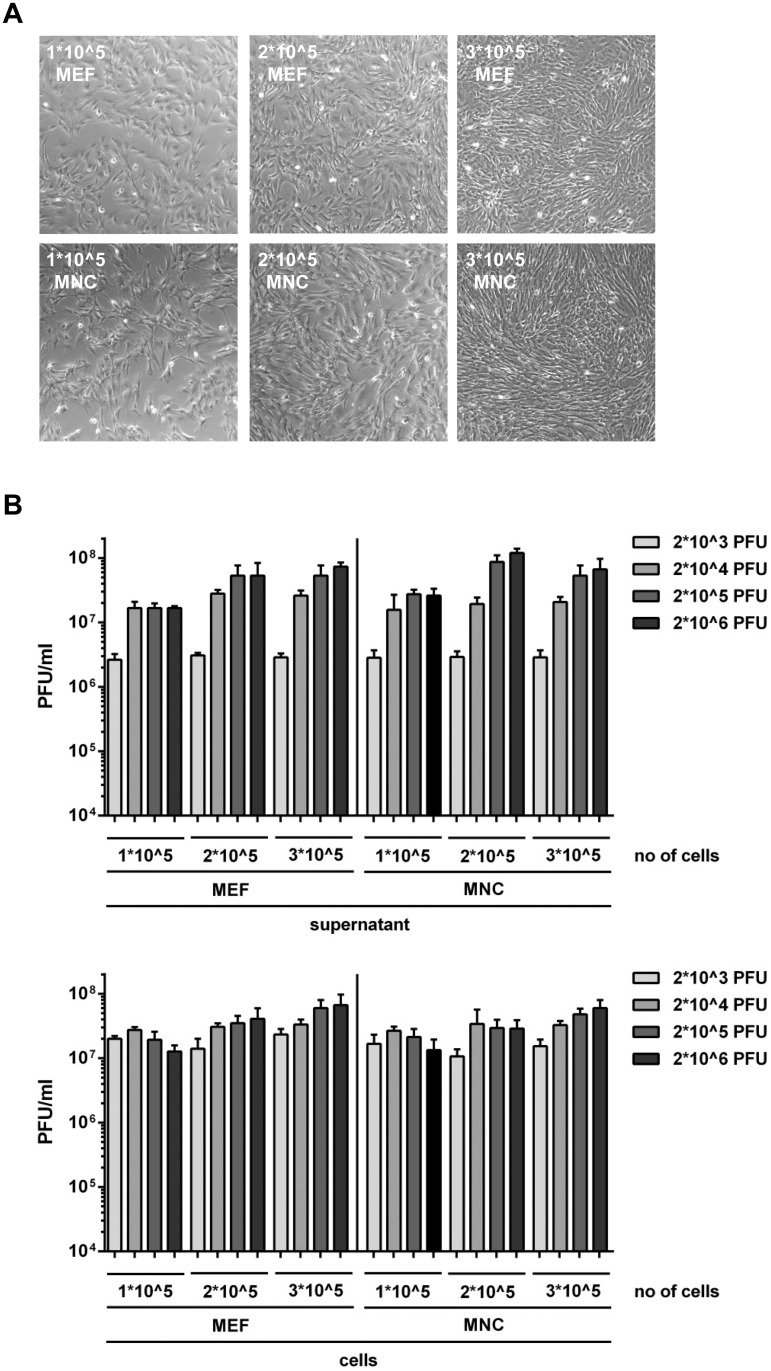
(A) 1*10^5, 2*10^5 or 3*10^5 of MEF and MNC, respectively, were seeded in 12 well plates. The confluency of the cells were monitored one day after seeding. (B) MEF and MNC from (A) were infected with indicated doses of MCMV. At 3 days post infection, supernatant and cell-associated virus was quantified by plaque titration on MEF. Each titration was done in triplicate. Mean values +/- SD are shown.

Since viral replication might differ depending on the cell densities and/or initial multiplicities of infection, we varied these conditions and analysed the viral replication in MEF and MNC. As shown in Fig 5, the MCMV titers depend on the density of the cells and the input dose of infection, but MEF and MNC are even under these different conditions highly similar.

### MNC can replace MEF for the generation of MCMV stocks and for plaque titrations

**Fig 6:**
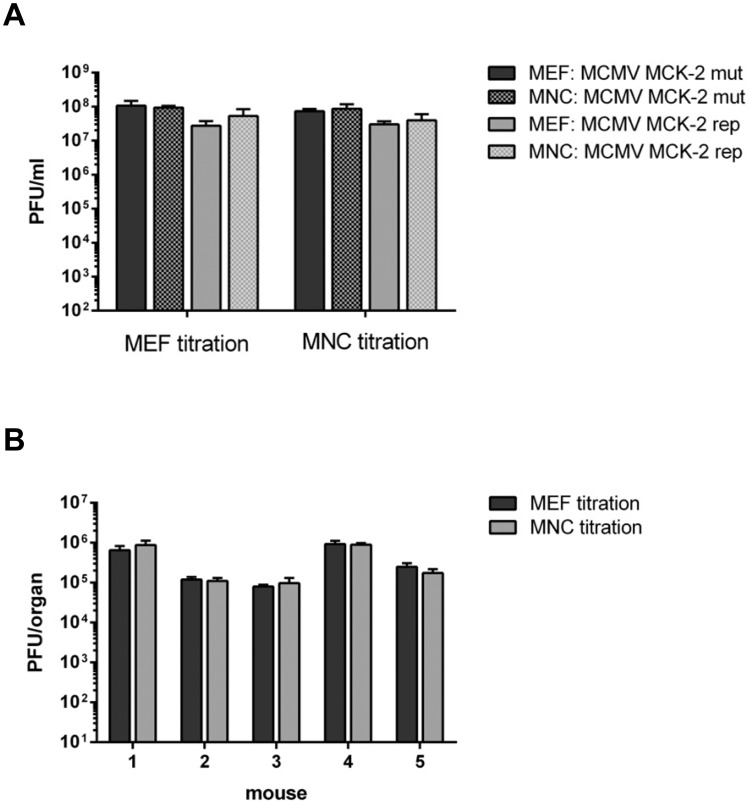
(A) MEF and MNC were used to generate virus stocks of MCK-2 mutated and MCK-2 repaired BAC-derived MCMV. The stocks were prepared by pooling the supernatant of completely infected cells and the cell homogenate. This virus suspension was cleared by centrifugation (3700 g for 10 min) before virus stock aliquots were frozen at -80°C. The stocks were titrated in triplicate on MEF and MNC in parallel. Mean values +/- SD are shown. (B) BALB/c mice were infected i.p. with 2*10^5 PFU of MCK-2 repaired BAC-derived MCMV. At 21 days post infection, the salivary glands were isolated and frozen. The virus titers were determined from organ homogenates by plaque titration on MEF and MNC in parallel. The titrations were done in quadruplicate. Mean values +/- SD are shown.

Since MEF are widely used for the generation of high titer virus stocks, we tested if MNC can also replace MEF in this respect. We generated stocks of two different viruses (BAC-derived MCMV MCK-2 mutated and repaired, respectively) in parallel on MEF and MNC. Both stock titers were then determined in parallel by plaque titration on MEF and MNC. The results demonstrated that MNC can be used for the generation of high titer virus stocks (Fig 6A), so that MNC can replace MEF in this high cell number consuming procedure. Additionally, MNC can be used for plaque titration of MCMV since the titers obtained from MEF and MNC were the same (Fig 6A). Several groups conduct MCMV experiments *in vivo*, therefore we wondered if MNC are applicable for determination of *in vivo* organ titers. To test this, we infected BALB/c mice with 2x10^5 PFU of MCK-2 repaired MCMV and harvested at 21 days post infection the salivary glands. The virus load of the organ homogenates was quantified by plaque titration on MEF and MNC, respectively. The values derived from MEF and MNC titrations were virtually identical (Fig 6B), demonstrating that MNC can be used for organ titrations.

### Identical IFNγ susceptibility of wt and ΔM27-MCMV in MEF and MNC

**Fig 7:**
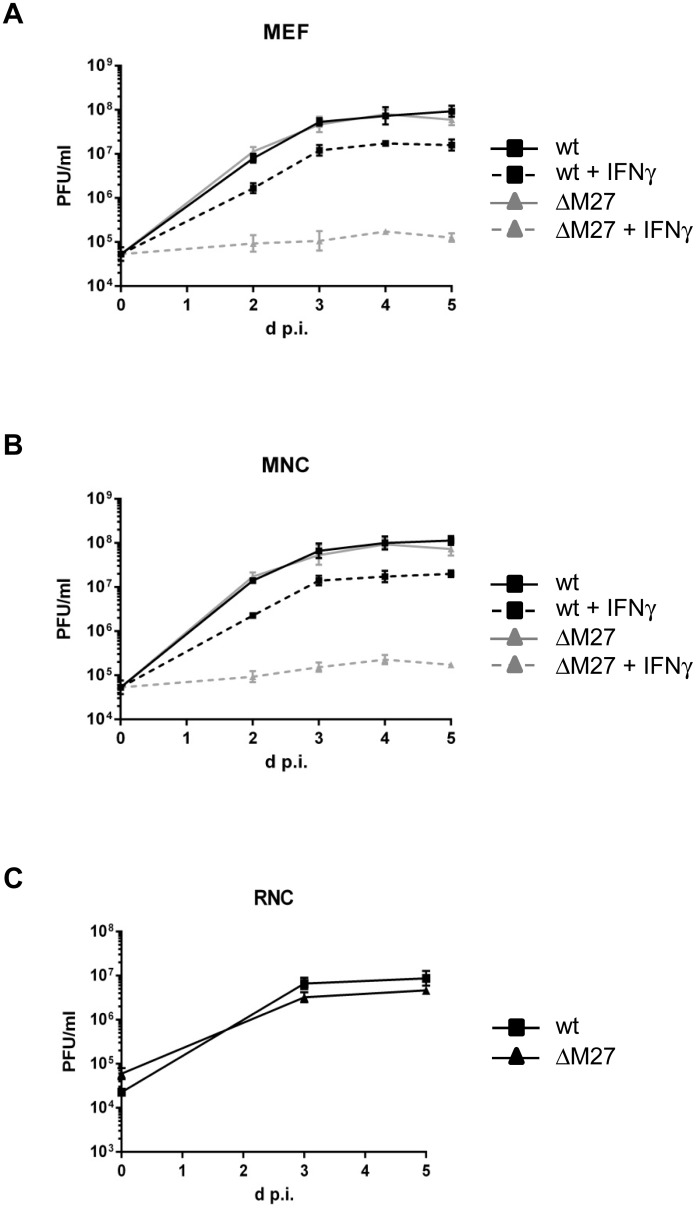
(A) MEF were left untreated or pre-incubated with 200 U/ml IFNγ before they were infected with wt-MCMV and ΔM27-MCMV, respectively. Virus titers were quantified after indicated time points. Each titration was done in triplicate. Mean values +/- SD are shown. (B) As in (A), but MNC were used. The MEF and MNC experiment was performed and titrated (plaque titration on MEF) in parallel. Each titration was done in triplicate. Mean values +/- SD are shown. (C) Rat newborn cells (RNC) were prepared and infected with wt-MCMV and ΔM27-MCMV, respectively. After 3 and 5 days post infection, cells were frozen. MCMV replication in RNC was analysed by plaque titration on MEF. Each titration was done in triplicate. Mean values +/- SD are shown.

To allow MCMV replication in the presence of interferon (IFN) and especially IFNγ, MCMV expresses the IFN antagonist pM27. In the absence of *M27* coding capacity, MCMV is highly susceptible to IFNγ [10,12]. To compare the IFN response of MEF and MNC, wt- and ΔM27-MCMV replication was studied in IFNγ-conditioned cells. While IFNγ had only limited antiviral potency against wt-MCMV in both cell types, ΔM27-MCMV replication was almost completely abrogated by IFNγ—again in both cells (Fig 7A and 7B). Taken together, this indicates that viral replication, the antiviral activity elicited by IFNγ and the phenotype of the deletion of a viral IFN antagonist are virtually identical in MEF and MNC.

### Rat newborn cells allow MCMV replication like rat embryonic fibroblasts (REF) do

One peculiar thing about the otherwise highly species specific MCMV replication is its ability to replicate in embryonic fibroblasts derived from rats (*Rattus norvegicus*)—conversely, rat CMV does not replicate in fibroblasts derived from mice [13,14]. To test the interchangeability of newborn cells and embryonic cells and to show their versatility beyond the species mouse, we prepared rat newborn cells (RNC) and infected them with wt-MCMV and ΔM27-MCMV. As shown in Fig 7C, both viruses efficiently replicate in rat newborn cells, indicating that even the species specificity is recapitulated in newborn cells.

## Discussion

Herein we describe a convenient protocol for the generation of adherent fibroblasts from newborn mice. We tested the suitability of these cells for cytomegalovirus research and observed that MNC fully recapitulate all tested aspects of the viral life cycle observed in MEF. In particular, the expression kinetics and abundance of *immediate early* (pIE1), *early* (pM45) and *late* (gB) proteins were highly similar. Viral replication as measured by a surrogate test (luciferase activity) and by classic plaque titration was almost identical in MEF and MNC. The susceptibility towards the antiviral drug GCV and the antiviral cytokine IFNγ was also congruent. Even aspects of species specificity, like the ability of mouse CMV to replicate in rat fibroblasts, were fully recapitulated in newborn cells.

The use of MNC has several advantages: (I) For the production of MEF, a gravid mouse has to be sacrificed for the preparation of embryos. The only alternative would be a caesarean section. This is not performed routinely and would be associated with pain, distress and suffering of the animal. When newborn mice are used instead of mouse embryos, there is obviously no need to sacrifice the dam. The dam can be used for further breeding or for other experiments. This aspect is especially relevant when expensive mouse lines or lines which do not breed well are used. (II) Newborn mice are larger than embryos and consistently yielded 2 to 4 times more cells (depending on the individual preparation) per sacrificed individual and/or pregnant mouse. (III) Uncertainty concerning gravidity which frequently leads to unnecessary killing of mice in the process of MEF production can easily be circumvented. Therefore, the use of MNC reduces the number of animals required for MCMV experiments and thereby satisfies the ‘reduce’ aspect of the 3R guiding principles. (IV) According to a recent amendment of the EU regulations concerning animal experiments, every experiment using pregnant mammals and/or the respective embryos in the last trimester of gestation explicitly requires a specific permission. An obvious but unethical work-around strategy would be to use younger embryos to which these regulations do not apply. However, this would further increase the number of animals required for research because younger foetus yield less cells. In addition, exactly terminated breeding is difficult to perform. Without proper side-by-side comparison between ‘early’ and ‘late’ MEF, such an approach might also be scientifically unsound because certain aspects affecting viral replication or innate immune responses might develop in this critical period of gestation. Changing the age of embryos used for MEF production might result in inconsistency with previous data sets. Obviously, MEF can still be used for MCMV research. However, scientists in the EU have to be aware that a specific permission to conduct such experiments is required. These strict regulations explicitly exclude animals which are killed (without pain) for the use of their organs or tissues. Scientists are allowed to prepare organs, tissues and the respective cells from newborn animals without a dedicated permission. Thus, the herein described approach provides a legal alternative for MEF production.

Besides above mentioned organisational, legal and animal experimental issues, we also feel that there is good scientific reason to use MNC instead of MEF. For scientific argumentation, findings derived from different model systems (e.g. *in vitro*, *ex vivo* and *in vivo*) are integrated. This connection is only valid, if the models are not different in relevant aspects. The main advantage of MCMV as compared to HCMV is the possibility to conduct *in vivo* experiments. MCMV is frequently studied in newborn as well as adult mice. In clear contrast to HCMV, a congenital infection from the pregnant dam to the developing foetus does not occur in the case of MCMV. Thus, embryos are not infected during typical *in vivo* experiments and it is at best risky to integrate *in vivo* findings obtained in newborn or adult mice with findings obtained from embryonic fibroblasts in cell culture.

In experiments which require the use of the F1 generation of a heterologous cross (e.g. to combine certain markers or alleles), our approach would allow a direct comparison between *in vitro* and *in vivo* experiments when the MNC are produced from littermates of the animals included in the *in vivo* experiment.

Taken together, MNC constitute a novel primary cell culture system which might not only be comparable but even superior to MEF culture in some important aspects.

## Materials and methods

### Viruses, infection conditions and virus titration

The BAC-derived MCMV mutants Δm157-MCMV:*dsRedMito*, Δm157-MCMV:*luciferase*, wt-MCMV and ΔM27-MCMV were described elsewhere [4,8,12]. Viral titers were determined by standard plaque titration [3] on MEF or MNC. All infections and titrations were done with centrifugal enhancement (800 g for 30 min). Ganciclovir (Sigma) was used at a concentration of 50 μM. Recombinant IFNγ was purchased from Merck Millipore. For the *in vivo* infection of BALB/c mice, a MCK-2 repaired BAC-derived MCMV [15] was used.

### Animal care

C57BL/6 and BALB/c mice were bred and housed in the animal facility of the Institute for Virology of the University Hospital Essen. Timed pregnant 10 to 20 weeks old female mice were used for generation of MEF. For MNC preparation, newborn mice were separated at day 1 to 2 after birth from the dam. For the generation of rat newborn cells, newborn Wistar unilever (HsdCpb: WU) rats were obtained from the central animal facility of the University Hospital Essen.

### Ethic statement

All procedures were done in accordance with EU regulations and with explicit permission of the local authorities (the *Landesamt für Natur*, *Umwelt und Verbraucherschutz*) in North Rhine Westphalia (#84–02.04.2014.A390 for MEF preparation and #84–02.04.2013.A414 for MCMV *in vivo* infection).

### Isolation of primary MEF and MNC

For MEF preparation, mouse embryos (day 16 to 17 postcoitum) were dissected into 10 ml sterile PBS in a 100 mm tissue culture dish. Embryonic internal organs were removed from the abdominal cavity. For MNC preparation, 1 to 2 days old newborn mice were sterilized in 70% (v/v) ethanol and thoroughly washed in PBS before the head, limbs and visceral organs were removed. After the dissection, the remaining steps of both MEF and MNC preparation were the same. The embryonic and newborn tissue was minced using 2 ml centrifuge tubes and scissors. The more homogeneous the minced tissue is, the better the yield of cells. The homogenate was transferred to a 50 ml conical tube (maximal 7 to 10 embryos and 3 to 4 newborn mice, respectively, per tube) and washed twice with PBS. The washed homogenate was incubated in 30 ml (per 50 ml conical tube) of cell culture grade trypsin solution (2.5% trypsin diluted 1:3 in sterile PBS) for 30 min at 37°C (e.g. in a cell culture incubator). 5 ml of 2.5% trypsin solution and 100 μl of DNaseI solution (10 mg/ml; Roche) were added before the homogenate was incubated for additional 60 min. 5 ml FCS was added to stop the trypsin reaction. In addition, the trypsin was removed by centrifugation for 5 min at 350 g and subsequent removal of the supernatant trypsin solution. The pellet was resuspended in 25 ml DMEM supplemented with 10% (v/v) FCS. A centrifugation step for 5 min at 350 g and subsequent removal of the supernatant medium was performed. The pellet was finally intensely resuspended in 50 ml growth medium. Large tissue pieces were allowed to settle to the bottom (5 to 10 min standing at room temperature). The cell solution was transferred to 175 cm^2^ cell culture flasks using 2 to 3 embryos and 1 newborn mouse, respectively, per flask. To increase the cell yield, a second round of intense resuspension of the remaining pellet can be performed. Cells were grown in DMEM supplemented with 10% (v/v) FCS, 100 μg/ml streptomycin, 100 U/ml penicillin and 2 mM glutamine until confluency (2 to 4 days). The medium was changed after the first day and cell density was monitored using an inverted microscope. Passage 0 cells were expanded 1:5 before passage 1 cells were frozen. For experiments, MEF and MNC were used in passage 3. All cell culture media and supplements were obtained from Gibco/Life technologies.

Rat newborn cells were generated following above mentioned MNC protocol.

### Luciferase assay

Luciferase activity was measured according to manufacturer's instructions (pjk) using a microplate luminometer (Mithras LB 943; Berthold).

### Immunoblot analysis

Immunoblotting was performed according to standard procedures (see e.g. [16]). Briefly, cells were lysed and equal amounts of protein were subjected to SDS-PAGE and transferred to nitrocellulose membranes. Immunoblot analysis was performed using the following antibodies: mAb anti-MCMV-IE1 (CROMA101), mAb anti-MCMV-M45 (described in [8]), mAb anti-MCMV-gB (15A12-H9) and anti-GAPDH (Santa Cruz). Proteins were visualized using peroxidase-coupled secondary antibodies and the ECL chemiluminescence system (New England Biolabs).
